# Correlates and Covariates of Type 2 Diabetes in an African American Population in the Washington DC Area

**DOI:** 10.4236/ojepi.2022.124035

**Published:** 2022-11

**Authors:** Jyothirmai J. Simhadri, Christopher A. Loffredo, Tanmoy Mondal, Zarish Noreen, Thomas Nnanabu, Ruth Quartey, Charles Howell, Brent Korba, Gail Nunlee-Bland, Somiranjan Ghosh

**Affiliations:** 1 Departments of Pediatrics and Child Health, College of Medicine, Howard University, Washington, DC 20059; 2 Department of Biology, Howard University, Washington, DC 20059; 3 Department of Oncology, Georgetown University, Washington, DC 20057; 4 Department of Microbiology & Immunology, Georgetown University, Washington, DC 20057; 5 Department of Healthcare Biotechnology, National University of Sciences and Technology (NUST), Islamabad 44000, Pakistan; 6 Viral Hepatitis Center, College of Medicine, Howard University, Washington DC 20059

**Keywords:** Diabetes, Epidemiology, Smoking, African American

## Abstract

In the United States, type 2 diabetes mellitus (T2DM) disproportionately affects the African American (AA) community, which has not been systematically included in molecular studies of underlying mechanisms. As part of a gene expression study, we recruited cases with T2DM and matched, unaffected controls at an urban hospital in Washington, DC, with a majority AA population. Here we describe the profile of socio-demographic, behavioral, and health-related associations of the study population. Self-reported data were collected from cases with T2DM (N=77) and age- and gender-matched controls (N=80), ages 45–65 years. Logistic regression was used to calculate odds ratios (OR) and 95% confidence intervals (CI). As expected, obesity, hypertension, and cardiovascular disease were more prevalent in cases than controls. Tobacco smoking and working alongside other tobacco smokers were also associated with T2DM. After adjusting for covariates, current tobacco smoking remained statistically associated with the disease (OR per half pack of cigarettes 1.43, 95% CI 1.04–1.95; *p*-value 0.027). HbA1c levels were elevated in T2DM cases who smoked more than a pack of cigarettes daily. These associations highlight the comorbid burdens of T2DM in an AA urban community setting and identify tobacco control as an unmet need for future prevention and control efforts.

## 1. Introduction

Type 2 diabetes mellitus (T2DM) is a chronic metabolic disorder that affects 463 million people worldwide, and this number is projected to increase to 642 million by 2040 [1]. It is estimated that in the United States alone, T2DM affects 34.2 million people (10.5% of the US population), with 26.9 million diagnosed and an estimated 7.3 million that remain undiagnosed [2]. Significant health disparities in T2DM and its complications and co-morbidities exist among racial/ethnic minorities in the U.S., both in terms of health outcomes and quality of care [3]. But persons from racial and ethnic minorities have not been systematically recruited into molecular studies of pathogenetic mechanisms of T2DM [4]. Since individuals with T2DM are at high risk for long-term complications [5], including hypertension, stroke, retinopathy, neuropathy, coronary artery disease, and end-stage renal disease, the identification of mechanisms and modifying factors is important for its prevention and control.

As part of an ongoing study of the Research Center in Minority Institutions Program at Howard University, we are conducting a gene expression-based investigation in African Americans with T2DM, enrolled at Howard University Hospital. We recruited adult cases with T2DM and matched, unaffected controls at this urban hospital setting, collected information through questionnaires and medical record abstraction that served as covariates and potential effect modifiers of the subsequent gene expression patterns. Here we describe the profile of socio-demographic, behavioral, and health-related associations of the study population.

## 2. Materials and Methods

### 2.1. Research Ethics Approval: Human Participants

The study was conducted with approval of the Howard University Institutional Regulatory Board (protocol number IRB-17-MED-44) (Supplementary File 1).

### 2.2. Study population

The eligible population consisted of adults aged 45–65 years old who self-identified as being AA. Persons with T2DM (cases, N=77) were recruited sequentially from the Diabetes Treatment Center at Howard University Hospital between October 2019 and April 2020. They were diagnosed according to American Diabetes Association criteria (https://www.diabetes.org/a1c/diagnosis) as follows: (a) fasting plasma glucose levels of 100 mg/dl (5.6 mmol/l) to 125 mg/dl (6.9mmol/l)], or (b) impaired glucose tolerance, as indicated by 2-h values in the oral glucose tolerance test of 140 mg/dl (7.8 mmol/l) to 199 mg/dl (11.0 mmol/l)], or (c) hemoglobin A1c (HbA1c) level of 5.7% or higher. Controls (N=80) were frequency-matched to the case group by gender and 5-year age group. They were persons without T2DM who were recruited in the Howard University Hospital cafeteria through special recruitment drive (which included both hospital staff and visitors).

### 2.3. Recruitment and Data Collection

The participants responded to an advertisement made through the Howard University Community Newsletter via email, flyers, through public announcements, and social media. A standardized questionnaire (Supplementary File 2) was used to collect information about education, economic status, smoking, alcohol consumption, and health history. Research participants filled in the paper forms under the supervision of the recruiter who assisted with any problems and checked for completeness. All participants provided signed informed consent. Medical records of cases were reviewed to confirm the T2DM diagnosis and to abstract the most recent HbA1c level.

### 2.4. Exclusion Criteria

The exclusion criteria for both cases and controls were having HIV/AIDS, cancer, or any major surgical procedure(s) in the last 5 years.

### 2.5. Measures

#### 2.5.1. Demographic factors

Present age was recorded at the time of questionnaire, and categorized into five groups: <50 years, 50–54 years, 55–59 years, 60–64 years, and ≥ 65 years.

#### 2.5.2. Socioeconomic factors

Socioeconomic factors included marital status, education, and employment. Marital status was based on 3 response categories i.e., married, never married, or previously married. Education was categorized as less than or equal to high school, 4 years of college, or a master’s degree or higher degree. Current employment status was categorized as either working or not working.

#### 2.5.3. Behavioral factors

Tobacco smoking history was ascertained as never smoked cigarettes, past smoker, or current smoker. Current tobacco smoking was grouped into the following categories: non-smoker, 1–10 cigarettes/day, 11–19 cigarettes/day, 20 cigarettes/day, and >20 cigarettes/day. We also asked about the number of years of living and working with smokers. Current alcohol consumption frequency was categorized as non-drinker, ≤ once per month, 1–2 times per month, once per week, 2–3 times per week, or “almost daily”.

#### 2.5.4. Medical history variables

Several variables that are known or suspected risk factors or co-outcomes of T2DM were ascertained in the questionnaire, including hypertension, cardiovascular disease, asthma, hay fever, and medication allergies, all of which were categorized as “yes” or “no”. Body mass index (BMI) was computed based on self-reported height and weight at the time of recruitment and categorized into three groups based on Centers for Disease Control and Prevention (CDC) cut-off points: low to normal weight (BMI = 18.50–24.99), overweight (BMI = 25.00–29.99), and obese (BMI > 30).

### 2.6. Statistical Analysis

Data were described using percentages or means and standard deviations (SD). In-dependent sample t-tests for continuous variables and chi-square tests for categorical variables were used to examine differences between cases and controls. We used multivariate logistic regression analyses to estimate the odds ratios [ORs], and 95% confidence intervals [CIs] for those variables that were statistically significant in the univariate analyses, and the matching factors of age and gender were included in the model. Statistical significance was set at *p* ≤0.05. All statistical analyses were performed using SAS, version 9.4.

## 3. Results

For the total participants, Table 1 lists the socio-demographic characteristics, comparing the cases and controls. The mean age of the study population at the time of enrollment was 56.3±7.70 and 56.8±6.01 years for the control and T2DM groups respectively, and the proportions of males and females were identical between the two groups, by design. As expected, given the recruited hospital staff members among the controls, there was a statistically significant association between T2DM and working status, with T2DM cases more likely than controls to report not working. In both the control and T2DM groups, the marital and educational status distributions were almost equal (p-values 0.14 and 0.90, respectively), and more than 60% of the recruited populations had a high school diploma or less education.

We observed that T2DM was associated with several medical conditions, including elevated BMI, hypertension, cardiovascular disease, and asthma, all of which were more prevalent in cases than controls (Table 2). Regarding BMI, the cases had a mean BMI of 36.8 (categorized as obese), whereas the mean BMI of the control group (29.2) was categorized as overweight. In the T2DM group, the mean age for the onset of T2DM was 43.6 years, with a mean HbA1c of 9.7% (83 mmol/mol). Among all these associations, BMI, hypertension, cardiovascular diseases were significantly elevated in the cases compared to controls (*p*-values < 0.05). Prevalence of hay fever and medication allergies was not significantly between cases and controls.

Table 3 shows the associations of tobacco use and alcohol consumption with T2DM. We observed a statistically significant association with current tobacco use (*p*=0.009) but not with alcohol consumption history (*p*=0.81). The number of years working with smokers was also associated with T2DM (*p*=0.03). The mean years of workplace secondhand smoke exposure was 12.2 years for the cases compared to 6.5 years for controls.

The results of the logistic regression model are shown in. Current tobacco smoking (OR=1.43 per half-pack, 95% CI=1.04–1.95, p-value 0.027) remained statistically associated with T2DM after adjustment for age and gender.

**Figure 1.**
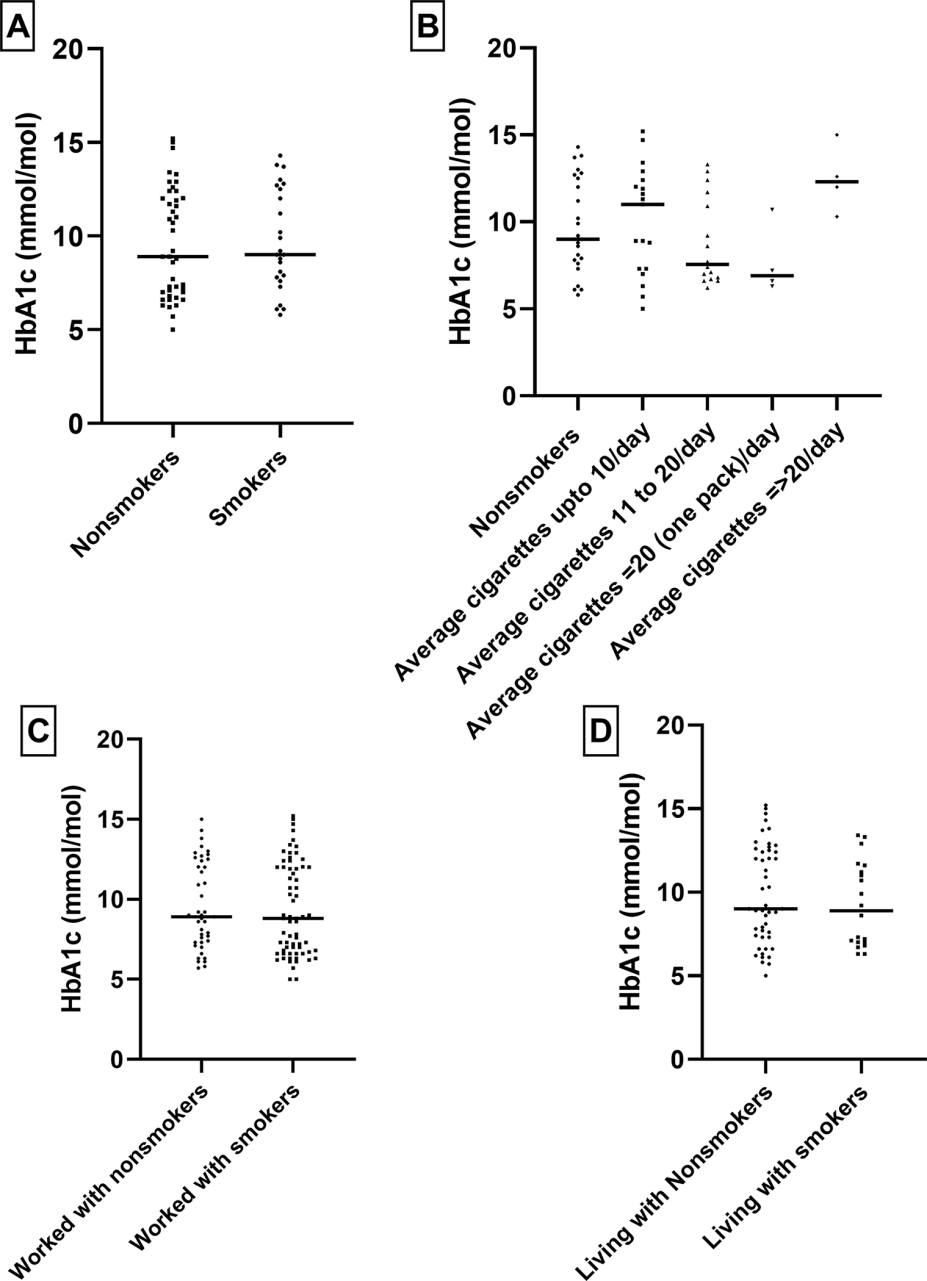
Relationship between HbA1C level and diabetic smokers/non-smokers population: To check any statistically significant relationship we performed T-test. No statistically significant relationship was observed.

**Figure 2.**
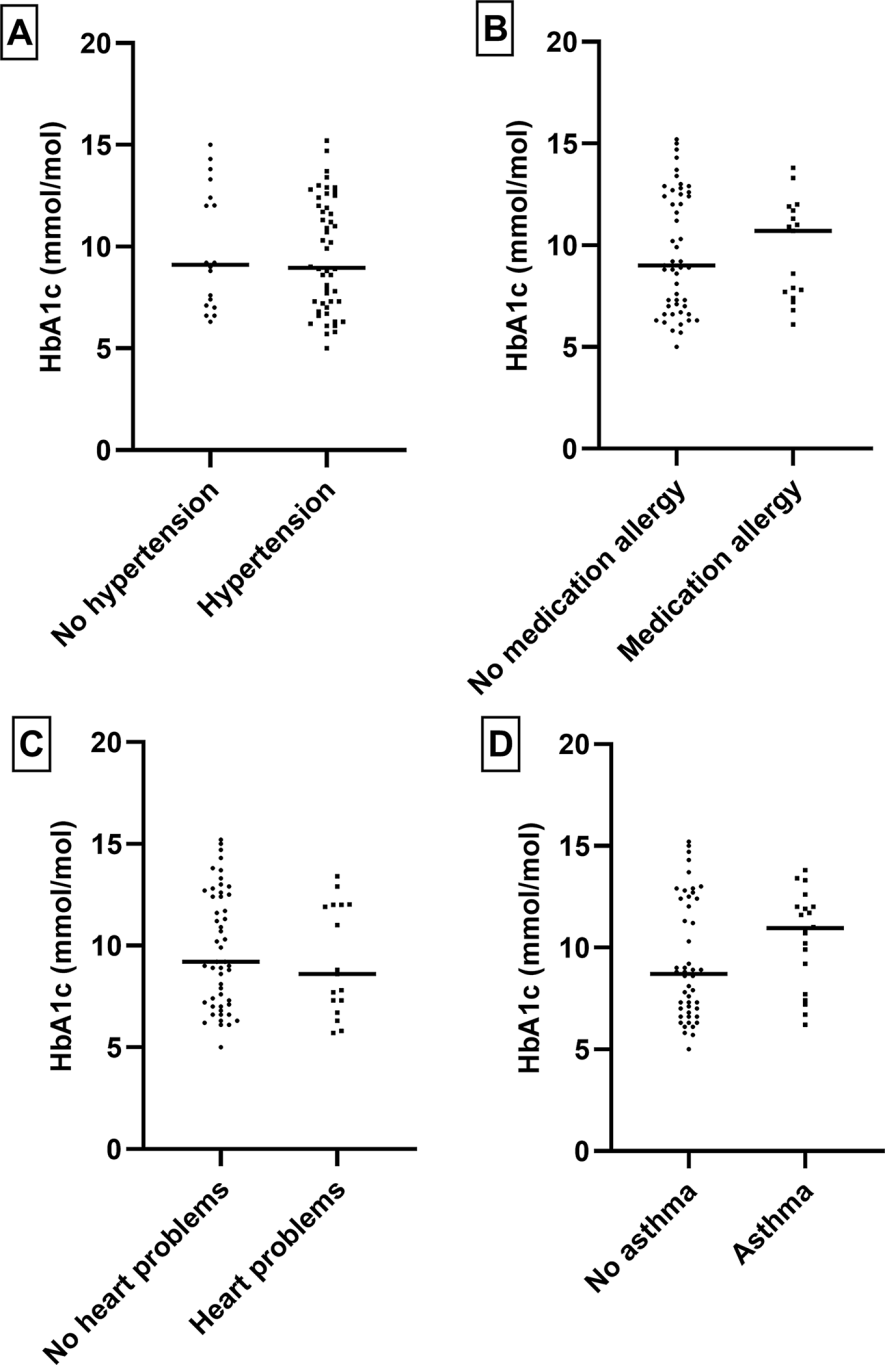
Relationship between HbA1C level and other type of factors e.g., Hypertension, Heart Problem, Asthma and Medication allergy in the diabetes population: To check any statistically significant relationship we performed T-test. No statistically significant relationship was observed.

**Figure 3.**
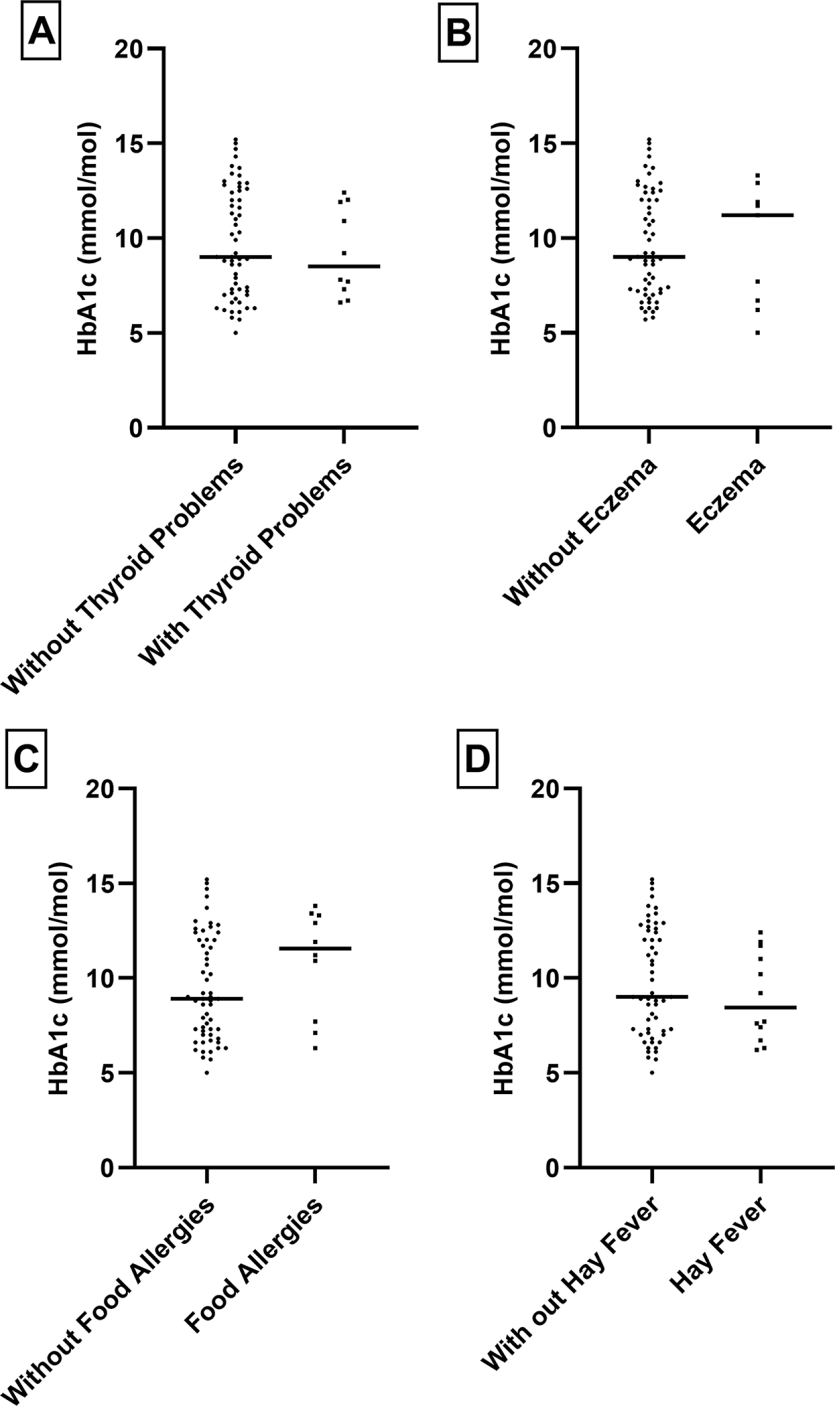
Relationship between HbA1C level and other different type of factors e.g., Thyroid problem, Eczema, Food Allergies and Hay Fever in the diabetes population: To check any statistically significant relationship we performed T-test. No statistically significant relationship was observed.

**Figure 4.**
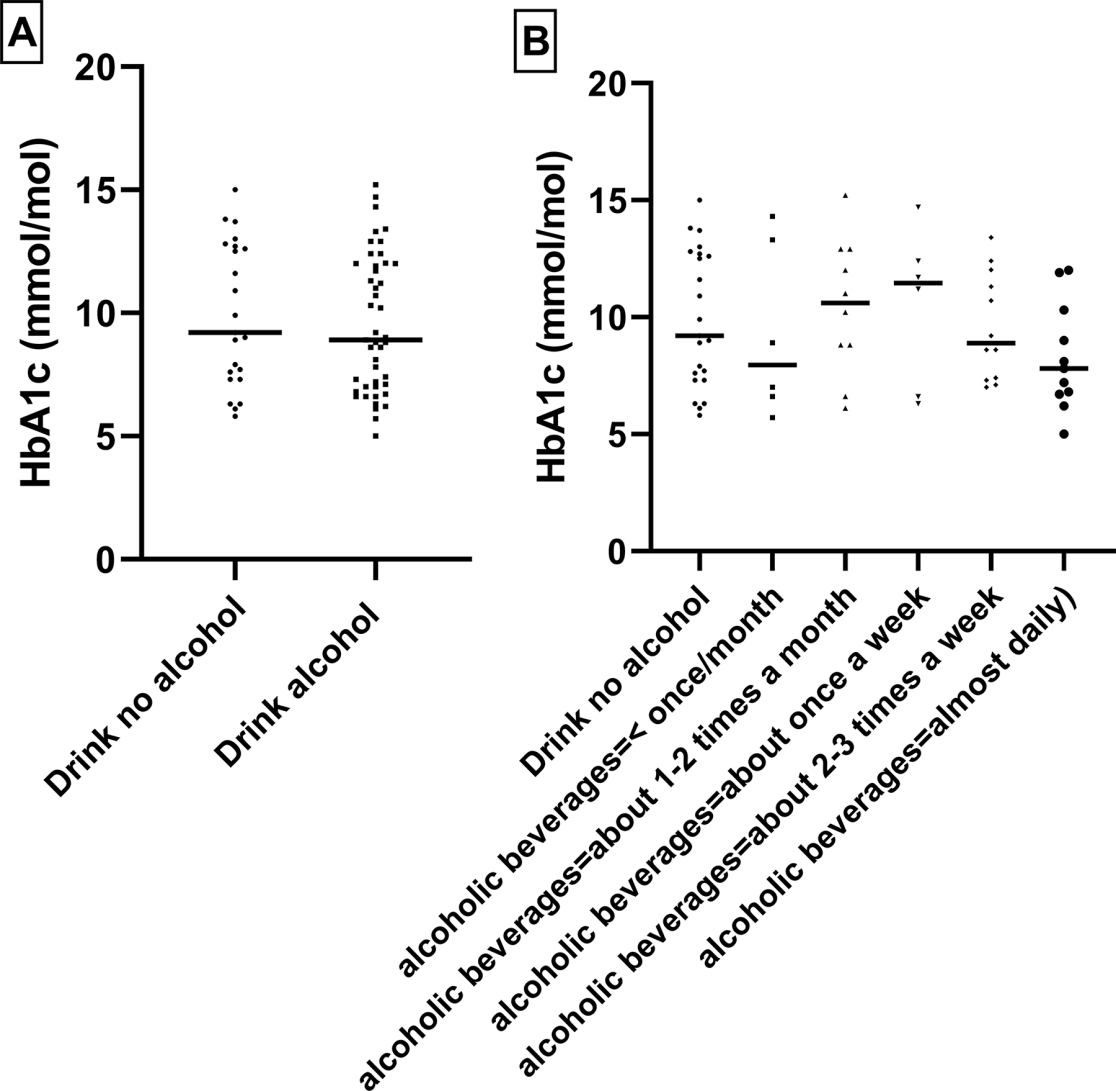
Relationship between HbA1C level and diabetes with alcohol drinking/ no alcohol drinking: To check any statistically significant relationship we performed T-test. No statistically significant relationship was observed.

Within the T2DM cases, we examined the patterns of HbA1c levels in relation to comorbidities and tobacco use and alcohol consumption, as shown in figure 1–4. Higher levels of this marker, suggesting suboptimal long-term glycemic control were observed in obese cases, those with hypertension, and those who smoked more than one pack of cigarettes per day.

## 3. Discussion

In this report, we examined the associations of demographic, socioeconomic, behavioral, and comorbid conditions of T2DM in an urban AA population. As expected, statistically significant associations were observed with extreme obesity and hypertension being much more common among the affected cases compared to age- and gender-matched controls. An unexpectedly strong association with current cigarette smoking was also observed, being much more prevalent among the cases than controls, and it also manifested with deleterious effects on the HbA1c levels of the cases.

Among the many health consequences, obese persons are more likely to develop T2DM (https://www.cdc.gov/healthyweight/effects/index.html). Our study design cannot determine whether obesity is a cause of consequence of T2DM. However, the association is consistent with other studies [6, 7], as T2DM and obesity are both associated with insulin resistance [8]. The META-Health Study of white and black residents aged 30–66 years living in the metro Atlanta area indicated that the average BMI in blacks was 31.4±7.6, consistent with what we observed in our Washington, DC-based study, where it was 36.8±11.4 in cases and 29.2±6.3 in controls [9]. According to recent maps of self-reported adult physical inactivity, there is 30.8% prevalence of physical inactivity in non-Hispanic black adults compared to 8% in whites in the District of Columbia area (https://www.cdc.gov/physicalactivity/data/inactivity-prevalencemaps/index.html), highlighting a possible intervention target for preventing T2DM.

It has been well established that T2DM contributes to the development of hypertension and other cardiovascular diseases, in relation to common underlying mechanisms of endothelial dysfunction, vascular inflammation, and dyslipidemia [10]. Since T2DM patients experience increased peripheral artery resistance, it causes elevated systemic blood pressure [11]. T2DM is also found to be associated with both macrovascular (involving large arteries such as conduit vessels) and microvascular dysfunctions (involving small arteries and capillaries) disease [12]. Echocardiography results in the Jackson cohort of the Atherosclerotic Risk in Communities (ARIC) study, which included middle-age black participants aged 45 to 64 years, revealed left ventricular hypertrophy (LVH) in 41% of Black women and 37% of Black men [13]. We also observed that, a large portion of T2DM patients in our AA cohort had hypertension and cardiovascular disease (*p*-values of 0.0001 and 0.03, respectively).

Regarding behavioral factors in our study, current tobacco smoking and years of exposure to secondhand smoke at the workplace were found to be associated with T2DM. Several prior reports indicated that smoking-induced inflammation may contribute to T2DM onset; however, the underlying mechanisms are still not known completely [14–16]. White et al [17] reported a study on a cohort of persons recruited from the tri-county area surrounding Jackson, MS, in the years 2000 to 2004, who were blacks aged 21 to 84 years. They found that AA who smoke more than 1 pack per day have a higher incidence of T2DM. The Insulin Resistance Atherosclerosis Study (IRAS) was another prospective study examining the relationship between smoking status and incident 5-year T2DM. They found that, of the current smokers, 25% developed T2DM at 5 years compared with 14% of never smokers. Similarly, we observed that the majority (64.8 %) of our study population was either a current or former smoker, and after multivariable adjustment, current smokers had a 2.66 times chance of exhibiting increased incident T2DM compared with never smokers [18].

Our study had some notable strengths and limitations. We recruited the study population from a single, urban hospital in Washington, DC, where cases and controls matched closely on age and gender. Cases had medically confirmed T2DM and patterns of associated comorbid conditions that were expected and consistent with prior research nationally. Controls were recruited from the same geographic area in which the cases re-sided. On the other hand, the recruitment of controls from among hospital staff meant that they were more likely than cases to be currently employed, to be healthier in general, and to report lower levels of tobacco smoking. While the modest sample size was sufficient to confirm the expected associations with comorbidities and to highlight additional environmental and behavioral associations, the statistical power to detect associations with less prevalent factors was limited.

## 5. Conclusions

In our cohort of AA men and women living in the urban Washington DC area, we found that patients with T2DM had higher levels of expected comorbid conditions such as hypertension and obesity, compared to controls. The unexpectedly high prevalence of tobacco smoking in the T2DM group and its extremely high levels of obesity suggest unmet clinical needs for smoking cessation and weight control interventions and treatments.

## Supplementary Material

Supplementary File -1

Suppementary File -2

## Figures and Tables

**Table 1.** Socio-demographic characteristics of the study population.

Characteristic	T2DM cases (n=77)			Controls (n=80)			

	n (%)	Mean	St. Dev.	n (%)	Mean	St. Dev.	P-value
Age (yrs.)		56.8	6.01		56.3	7.7	0.65
Age group:							
< 50	9 (11.7)			10 (12.5)			0.99
50–54	19 (24.7)			19 (23.8)			
55–59	19 (24.7)			20 (25.0)			
60–64	20 (26.0)			21 (26.2)			
65+	10 (12.9)			10 (12.5)			
Gender:							
male	37 (48.0)			41 (51.3)			0.69
female	40 (52.0)			39 (48.7)			
Occupation:							
Not working	48 (62.3)			35 (43.7)			0.02
Working	29 (37.7)			45 (56.2)			
Marital status:							
Married	22 (28.6)			22 (27.5)			0.14
Never married	41 (53.2)			33 (41.2)			
Previously married	14 (18.2)			25 (31.3)			
Education:							
≤ high school	45 (66.2)			45 (60.8)			0.90
4 years of college	14 (20.6)			17 (23.0)			
MS or higher degree	9 (13.2)			12 (16.2)			

Note: p-values from T-tests (continuous variables) or chi-square tests (categorical variables)

**Table 2.** Medical history variables in cases and controls.

Characteristic	T2DM cases (n=77)			Controls (n=80)			

	n (%)	Mean	St. Dev.	n (%)	Mean	St. Dev.	*P*-value
Body mass index		36.8	11.4		29.2	6.3	<0.0001
BMI group:							
< 25	12 (15.6)			29 (36.2)			
25–20	13 (16.9)			23 (28.8)			
30+	52 (67.5)			28 (35.0)			0.0002
Age at onset of diabetes (cases)		43.6	11.4				
HbA1c level (cases)		9.7	2.8				
Hypertension							
No	19 (24.7)			53 (68.8)			<0.0001
Yes	58 (75.3)			24 (31.2)			
Cardiovascular disease:							
No	57 (77.0)			70 (89.7)			0.03
Yes	17 (23.0)			8 (10.3)			
Asthma							
No	56 (77.8)			71 (88.7)			0.07
Yes	16 (22.2)			9 (11.3)			
Hay fever							
No	63 (86.3)			69 (88.5)			0.69
Yes	10 (13.7)			9 (11.5)			
Medication allergies							
No	59 (80.8)			62 (82.7)			0.77
Yes	14 (19.2)			13 (17.3)			

Note: p-values from T-tests (continuous variables) or chi-square tests (categorical variables)

**Table 3.** Tobacco and alcohol consumption in cases and controls.

Characteristic	T2DM cases (n=77)			Controls (n=80)			

	n (%)	Mean	St. Dev.	n (%)	Mean	St. Dev.	*P*-value
Current tobacco smoking:							
Non-smoker	27 (35.0)			41 (51.3)			0.007
Former smoker	27 (35.0)			14 (17.5)			
Current smoker	23 (29.8)			25 (31.2)			
1–10 cigarettes/day	25 (32.5)			27 (33.7)			
11–19 cigarettes/day	17 (22.1)			4 (5.0)			
20 cigarettes/day	4 (5.2)			7 (8.8)			
>20 cigarettes/day	4 (5.2)			1 (1.2)			
Years since quitting		10.8	11.1		16.3	10.2	0.14
Living with smokers							
No	51 (66.2)			55 (68.8)			0.74
Yes	26 (33.8)			25 (31.2)			
Years living with smokers		13.1	11.6		9.2	11.7	0.23
Working with smokers:							
No	49 (63.6)			50 (62.5)			0.88
Yes	28 (36.4)			20 (37.5)			
Years working with smokers		12.2	11.4		6.5	7.9	0.03
Current alcohol drinking:							
Non-drinker	24 (31.2)			33 (41.3)			0.81
≤ once per month	9 (11.7)			8 (10.0)			
1–2 times per month	12 (15.6)			8 (10.0)			
Once per week	7 (9.0)			6 (7.5)			
2–3 times per week	13 (16.9)			13 (16.2)			
Almost daily	12 (15.6)			12 (15.0)			
Years of alcohol drinking		15.9	11.3		18.4	13.1	0.34

Note: p-values from T-tests (continuous variables) or chi-square tests (categorical variables)

## Data Availability

The authors declare no conflict of interest. Data are stored and may be available upon reasonable request complying with the current data sharing policy of NIH, available at https://grants.nih.gov/grants/guide/notice-files/NOT-OD-03-032.html. The data sets used in this study include personal information. Thus, datasets are available from the corresponding author, Dr. Somiranjan Ghosh, on reasonable requests.

## References

[1] Atlas ID (2019) International Diabetes Federation. 9th edition ed. Brussels, Belgium: Federation ID.

[2] Centers for Disease Control and Prevention. (2020) National Diabetes Statistics Report. Atlanta, GA: Services USDoHaH. U.S.

[3] Department of Health and Human Services. (2015) Centers for Medicare & Medicaid Services. Baltimore: Office of Minority Health HDitMP.

[4] Galicia-Garcia U, Benito-Vicente A, Jebari S, Larrea-Sebal A, Siddiqi H, Uribe KB, … Martín C (2020) Pathophysiology of Type 2 Diabetes Mellitus. International Journal of Molecular Sciences, 21(17), 6275. doi:10.3390/ijms21176275 32872570 PMC7503727

[5] Melmed S, Auchus R, Goldfine A, Koenig R, Rosen C (2020) editors. Williams Textbook of Endocrinology. Vol. Chap 37. Philadelphia, PA: Elsevier.

[6] West DS, Dutton G, Delahanty LM, Hazuda HP, Rickman AD, Knowler WC, Vitolins MZ, Neiberg RH, Peters A, Gee M, Cassidy Begay, M., Look ARG (2019) Weight Loss Experiences of African American, Hispanic, and Non-Hispanic White Men and Women with Type 2 Diabetes: The Look AHEAD Trial. Obesity. (Silver Spring). 27(8), 1275–1284. doi:10.1002/oby.22522. 31338998 PMC6658112

[7] Barnes AS (2011) The epidemic of obesity and diabetes: trends and treatments. Tex. Heart. Inst. J. 38(2), 142–144. 21494521 PMC3066828

[8] Al-Goblan AS, Al-Alfi MA, Khan MZ (2014) Mechanism linking diabetes mellitus and obesity. Diabetes. Metab. Syndr. Obes. 7, 587–591. 25506234 10.2147/DMSO.S67400 PMC4259868

[9] Hendley Y, Zhao L, Coverson DL, Din-Dzietham R, Morris A, Quyyumi AA, Gibbons GH; & Vaccarino V (2002) Differences in weight perception among blacks and whites. J. womens. health. (Larchmt). 20(12), 1805–1811. 10.1089/jwh.2010.2262. PMC3236990 21988528

[10] Cheung BMY, Li C (2012) Diabetes and hypertension: is there a common metabolic pathway? Curr. Atheroscler. Rep 14(2), 160–166. doi:10.1007/s11883-012-0227-2. 22281657 PMC3314178

[11] Petrie JR, Guzik TJ, Touyz RM (2018) Diabetes, Hypertension, and Cardiovascular Disease: Clinical Insights and Vascular Mechanisms. Can. J. Cardiol. 34(5), 575–584. doi:10.1016/j.cjca.2017.12.005. 29459239 PMC5953551

[12] Brownlee M (2005) The Pathobiology of Diabetic Complications. Diabetes. 54(6), 1615. doi:10.2337/diabetes.54.6.1615. 15919781

[13] Fox E, Taylor H, Andrew M, Han H, Mohamed E, Garrison R, Skelton T (2004) Body Mass Index and Blood Pressure Influences on Left Ventricular Mass and Geometry in African Americans. Hypertension. 44(1), 55–60. doi:10.1161/01.HYP.0000132373.26489.58. 15184348

[14] Effoe VS, Correa A, Chen H, Lacy ME, Bertoni AG (2015) High-Sensitivity C-Reactive Protein Is Associated With Incident Type 2 Diabetes Among African Americans: The Jackson Heart Study. Diabetes. care. 38(9), 1694–1700. doi:10.2337/dc15-0221. 26068864 PMC4542275

[15] Tuovinen E-L, Saarni SE, Männistö S, Borodulin K, Patja K, Kinnunen TH, Kaprio J, Korhonen T (2016) Smoking status and abdominal obesity among normal- and overweight/obese adults: Population-based FINRISK study. Prev. Med. Rep. 4, 324–330. doi:10.1016/j.pmedr.2016.07.003. 27486563 PMC4959936

[16] Wellen KE, Hotamisligil GS (2005) Inflammation, stress, and diabetes. J. Clin. Invest. 115(5), 1111–1119. doi:10.1172/JCI25102. 15864338 PMC1087185

[17] White WB, Cain LR, Benjamin EJ, DeFilippis AP, Blaha MJ, Wang W, Okhomina V, Keith RJ, Al, Rifai. M., Kianoush S, Winniford MD, Robertson RM, Bhatnagar A, Correa A, Hall ME (2018) High-Intensity Cigarette Smoking Is Associated With Incident Diabetes Mellitus In Black Adults: The Jackson Heart Study. J. Am. Heart. Assoc. 7(2), e007413. doi:10.1161/JAHA.117.007413. 29330255 PMC5850161

[18] Foy CG, Bell RA, Farmer DF, Goff DC, Wagenknecht LE (2005) Smoking and Incidence of Diabetes Among U.S. Adults. Diabetes. Care. 28(10), 2501. doi:10.2337/diacare.28.10.2501. 16186287

