# Supplementary material for: Correlates and Covariates of Type 2 Diabetes in an African American Population in the Washington DC Area
Source: Open J Epidemiol. Author manuscript; Available in PMC 2024 Aug 2. (PMC11296657; doi:10.4236/ojepi.2022.124035)
Supplement: Supplementary File -1 [file NIHMS2008379-supplement-Supplementary_File_-1.pdf]

HOWARD UNIVERSITY

Office of Regulatory Research Compliance

Date: October 9, 2019

To: Somiranjan Ghosh, PhD.
Department of Biology

From: The Office of Regulatory Research Compliance

Title: **IRB-17-MED-44:** Validating a Gene Expression Signature for Type 2 Diabetes in a Low Environmental Exposure Setting of African American Population

Approval Date: October 9, 2019

Expiration Date: April 9, 2020

Action: Full Board Review- *Continuation: Active; Still Enrolling Participants*

The above-referenced submission was **approved** by the Institutional Review Board during the October 9, 2019 meeting. Approval for this study is through **April 9, 2020**

Please be reminded of the following:

1. It is your responsibility to ensure that a continuing review report is submitted to the IRB in a timely manner. Should you anticipate renewing this protocol at the end of the approved time frame, please submit the A-2 Form **60 days prior to the expiration date** (Please note that this office will automatically terminate the project on the date stated above, unless reviewed and re-approved by the IRB.);
2. If you plan to close this protocol, a close-out report must be submitted to the IRB within 30 days after completion. Use an A-2 Form for this purpose as well; and
3. During the project period of this research, the IRB has the right to conduct a monitoring site visit and you will be given prior notice.
4. IRB date-stamped consent documents should be used when obtaining informed consent;
5. All informed consent documents must be kept on record with this project and should be archived by you for at least three (3) years after the last date of the IRB approval; and
6. Any changes including changes in personnel, modifications to the protocol and advertising must be reviewed and approved by the IRB prior to initiation.
7. The HU IRB Federal Wide Assurance number is FWA00000891.

Please refer to the above mentioned date and protocol number when making inquiries concerning this protocol.

CC: IRB File
Thomas O. Obisesan, M.D., MPH, F.A.A.F.P., AVP of Regulatory Research Compliance
Marline Brown-Walthall, MPH, Sr. Compliance Administrator

HU Research Building 1
1840 Seventh Street, NW, Suite 309
Washington, DC 20001

(T) 202 865-8597
Fax (202) 232-5286
www.howard.edu
