# Supplementary material for: Correlates and Covariates of Type 2 Diabetes in an African American Population in the Washington DC Area
Source: Open J Epidemiol. Author manuscript; Available in PMC 2024 Aug 2. (PMC11296657; doi:10.4236/ojepi.2022.124035)
Supplement: Suppementary File -2 [file NIHMS2008379-supplement-Suppementary_File_-2.pdf]

Developing a gene expression signature for type 2 diabetes in a low environmental exposure setting

Howard University Hospital, 2041 Georgia Ave NW, Washington, DC 20059
Lead Investigator: Dr. Somiranjan Ghosh, PhD (PI/PD)
Dr. Christopher A. Loffredo, PhD (PI/PD, *MPI*)
Dr. Gail Nunlee-Bland, MD (PI/PD, *MPI*)
Contact Tel: (202) 806-4176 (Ghosh); (202) 687-3758 (Loffredo); 202-865-4758 (Nunlee-Bland)
e-mail: sghosh@Howard.edu

Questionnaire

Interviewer's Instructions:

Please use a ballpoint pen. If you make a mistake, cross out the incorrect answer instead, and rewrite the correct response, even if it is beside a space.

There are four kinds of questions:

(1) **blank line**: please write legibly using capital letters.

(2) **Where date is needed** - for month, use its number; for the year, use four digits. For example: May 16, 016 will be: 5/16/2016

(3) **Check boxes** – please use an X to mark your selection

(4) **Typed possible answers** (for example, YES/NO) or listed numbers: circle your selection.

If you leave a space blank, we will think that you have forgotten to fill in the answer, and we will need to contact you again to clarify any issues.

APPROVED
Howard University IRB
EXPIRES
APR 09 2020

Section A. Personal Data

1. Surname and first name: ____________________
2. Date of birth : ____________________

3. Current marital status:
   - ☐ married, or living as if married
   - ☐ single (never married)
   - ☐ widowed
   - ☐ divorced
   - ☐ permanently separated

4. Address :

   Street and number ____________________
   Town ____________________
   Postal Code ____________________
   Phone number at Home ____________________
   Mobile phone number ____________________
   Phone number at Work ____________________

APPROVED
Howard University IRB
EXPIRES
APR 09 2020

5. Gender ☐ Male ☐ Female

6. What is your present occupation / employment status? ______________________

7. What is the highest level of education you have completed?
   - ☐ High school or under
   - ☐College BS
   - ☐MS
   - ☐Ph.D.
   - ☐Other Professional Degree
   - ☐other __________ specify

Section B. Smoking History

We are asking for information about you and all persons who are ***CURRENTLY*** *living together with you, regarding tobacco smoke .*

1. Have you ever smoked in your life for longer than 3 months? YES/NO (if NO, skip to Q.4)

2. For how many years were you smoking regularly? ____ *(years)*
a. How much do you, or did you, smoke, in terms of average cigarettes per day:
   <10 (less than a half-pack)
   10-20 (a half-pack to a whole pack)
   >20 (more than one pack)

3. Are you currently smoking? YES/NO
a. If not, how many years ago did you quit? _____*(years)*

4. Are you living in a home where there are smokers? YES/NO
a. If YES, for how long? _____ *(years)*

5. Have you ever worked in a common room with smokers? YES/NO
a. If YES, for how long? _____ *(years)*

Section C. Alcohol Consumption

1. Did you ever drink alcohol regularly in your life? YES/NO (if NO, go to Section D)
2. For how many years were you drinking alcohol regularly? ____ *(years)*
3. On average, how often do you, or did you, have alcoholic beverages?
   < once per month
   about 1-2 times a month
   about once a week
   about 2-3 times a week
   almost daily

APPROVED
Howard University IRB
EXPIRES
APR 09 2020

4. **What kinds of alcoholic beverages do you, or did you, consume?**
(a) Beer YES/NO. If Yes, how many at one time? _______ (*number*)
(b) Wine YES/NO. If Yes, how many at one time? _______ (*number*)
(c) Liquor YES/NO. If Yes, how many at one time? _______ (*number*)

Section D. General Health

(*interviewer to measure height and weight at the clinic*)

Current Weight: ___ pounds
Height: ____ feet ____ inches
Did a doctor ever tell you that you had:

Question #	Health condition	YES	NO	DON'T KNOW	If Yes, from what age?
1	Diabetes				
2	Hypertension (high blood pressure)				
3	Thyroid problems				
4	Heart problems				
5	Eczema				
6	Food allergies				
7	Asthma				
8	Hay fever				
9	Medication allergy or adverse reaction				

10. Have you had any mold or mildew inside your home, in the last 5 years?

☐ Yes ☐ No ☐ Don't know

Section E. Adminstrative Information

1. Name of the Person Completing the Form (please print name): ______________________
2. Signature of the person completing the Form: ______________________
3. Date questionnaire completed: ______________________

APPROVED
Howard University IRB
EXPIRES
APR 09 2020
